# 3D-Printable Photothermal and Temperature-Controlled Polycaprolactone Scaffolds Incorporating Gold Plasmonic Blackbodies for Bone Tissue Engineering

**DOI:** 10.1021/acsami.5c05707

**Published:** 2025-05-13

**Authors:** Chieh-Ying Chen, Ruaina Lily Hope Gadia Moreno, Po-Yao Wang, Thanh Sang Nguyen, Jia-Lin Wu, Kuan-Hao Chen, Chih-Hwa Chen, Chia-Ying Lin, Pei-Chun Wong

**Affiliations:** † Graduate Institute of Biomedical Optomechatronics, College of Biomedical Engineering, 38032 Taipei Medical University, Taipei 11031, Taiwan; ‡ School of Biomedical Engineering, College of Biomedical Engineering, 38032 Taipei Medical University, Taipei 11031, Taiwan; § Graduate Institute of Biomedical Materials and Tissue Engineering, College of Biomedical Engineering, Taipei Medical University, Taipei 11031, Taiwan; ∥ Department of Orthopedics, 63474 Taipei Medical University Hospital, Taipei 11031, Taiwan; ⊥ International Ph.D. Program in Medicine, College of Medicine, 243733 Taipei Medical University, Taipei 11031, Taiwan; # Department of Trauma, Hue Central Hospital, Hue 530000, Vietnam; ∇ Department of Orthopedics, School of Medicine, College of Medicine, 38032 Taipei Medical University, Taipei 11031, Taiwan; ○ Orthopedics Research Center, Taipei Medical University Hospital, Taipei 11031, Taiwan; ◆ Centers for Regional Anesthesia and Pain Medicine, Wan Fang Hospital, Taipei Medical University, Taipei 11096, Taiwan; ¶ Department of Orthopedics, Shuang Ho Hospital, Taipei Medical University, New Taipei 23561, Taiwan; †† Convergent Bioscience and Technology Institute, Department of Biomedical Engineering and Informatics, 1772 Indiana University, Indianapolis, Indiana 46202, United States

**Keywords:** 3D printing, polycaprolactone, gold plasmonic blackbody, photothermal effect, NIR laser irradiation

## Abstract

Three-dimensional (3D) printing technology has revolutionized the design and fabrication of bone scaffolds, offering precise and customizable solutions for bone tissue engineering. In this study, we developed polycaprolactone (PCL) scaffolds that incorporated gold plasmonic blackbodies (AuPBs) to harness photothermal properties for temperature-controlled bone regeneration. The AuPB–PCL scaffolds demonstrated enhanced mechanical strength, a tunable thermal response under near-infrared (NIR) laser irradiation, and improved osteogenic potential. Photothermal stimulation effectively modulated cellular responses, promoting osteoblast proliferation, alkaline phosphatase (ALP) activity, and mineralization. Notably, mild hyperthermia (39–41 °C) induced by laser irradiation optimized osteogenesis, while excessive temperatures (≥42.5 °C) impaired cellular function due to mitochondrial stress and oxidative damage. These findings highlight the potential of AuPB–PCL scaffolds for controlled photothermal bone regeneration, offering a promising strategy for precise, completely noninvasive stimulation of bone repair.

## Graphical abstract

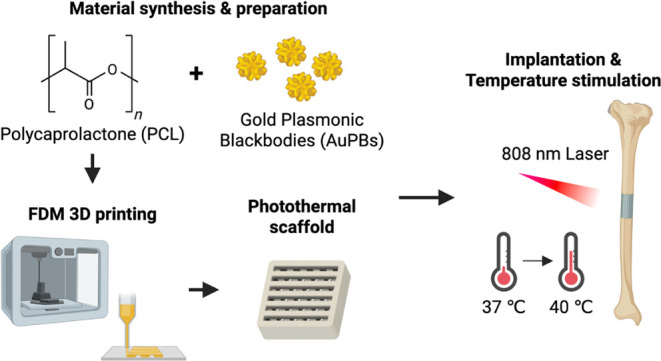

## 1 Introduction

Bone defects from tumors, deformities, injuries, and infections affect millions annually, with over two million bone grafts performed globally each year.^1^ Autologous grafts are the “gold standard” for repair but are limited by donor site morbidity and pain.^2^ Allogeneic grafts, while more available, carry risks of immune rejection and lower osteogenic potential.^3^ Repairing large defects remains challenging due to delayed or failed healing.^4^ Moreover, the irregular and complex shapes of bone fractures make it difficult to fabricate precisely fitting implants using traditional machining methods.^5^

Bone tissue engineering offers a promising solution by integrating seed cells, growth factors, and scaffolds.^6^ Scaffolds play a key role in mimicking the extracellular matrix (ECM), supporting cell growth, and promoting differentiation. Ideal scaffolds provide biocompatibility, osteoconductivity, and controlled degradability while maintaining mechanical strength.^7,8^ These properties enable effective regeneration of critical-size defects and allow customization of implants to fit complex geometries, addressing the limitations of traditional grafts and fabrication methods.

Bone remodeling is a dynamic and continuous process, with temperature variations being considered to be one of the key factors influencing this process. According to Allen’s Rule and its hypothesis on thermoregulation, animals in warmer environments tend to have longer limbs, indirectly supporting the association between temperature and bone growth.^9^ The biological performance of bone tissue significantly varies under different temperature conditions: temperatures below 36.6 °C suppress osteogenesis due to insufficient heat; temperatures between 36.6 and 37.5 °C maintain a balance between bone formation and resorption; and mild heat shock temperatures in the range of 39–41 °C enhance the function of osteoblasts and osteocytes, thereby promoting bone tissue formation. However, when temperatures exceed 42 °C, in the sublethal heat shock zone, bone growth significantly slows, and temperatures above 50 °C in the non-critical shock zone can induce osteocyte death.^10^ Since cancellous bone is more sensitive to high temperatures, it is crucial to precisely control the temperature within the range of 39–42 °C when applying temperature to promote bone repair.^11^

Heat shock also triggers a series of physiological responses. The mild heat shock zone activates the membrane enzyme adenylyl cyclase and cAMP-dependent protein kinase (protein kinase A). The latter can phosphorylate various heat-shock-related proteins (e.g., HSP70) and metabolic enzymes, further enhancing the activity of alkaline phosphatase (ALP) and promoting the calcification process of bone tissues. Additionally, heat shock increases the synthesis of insulin-like growth factor (IGF)-1 and other growth factors, enhances blood circulation and cell division, and further promotes osteoblast proliferation and bone tissue regeneration.^10^

Three-dimensional (3D) printing has emerged as a transformative technology in orthopedic applications, particularly in the design and fabrication of scaffolds for bone repair and regeneration.^12–14^ The primary goal of 3D-printed scaffolds in orthopedics is to mimic the native ECM of bone tissues, providing a structural framework that supports cell attachment, proliferation, and differentiation.^15,16^ These scaffolds must possess key properties such as biocompatibility, mechanical strength, and controlled porosity to facilitate effective nutrient transport, vascularization, and waste removal, which are essential for tissue regeneration.^17,18^ Additionally, the ability to customize scaffold designs to match patient-specific anatomical defects enhances the precision and efficacy of orthopedic treatments.^19^ This level of customization, combined with the potential for incorporating bioactive molecules, growth factors, or nanoparticles (NPs), makes 3D-printed scaffolds a critical tool in addressing challenges such as nonunion fractures, large bone defects, and degenerative bone diseases. By enabling the creation of scaffolds tailored to individual needs, 3D printing has the potential to revolutionize regenerative medicine and improve outcomes in orthopedic surgery.

Polycaprolactone (PCL) has become a widely used material in the development of 3D-printed scaffolds for orthopedic applications due to its unique combination of properties.^20,21^ PCL is a biocompatible and biodegradable polymer with a slow degradation rate, making it particularly suitable for bone regeneration, where prolonged scaffold stability is required to support tissue growth and remodeling.^22^ Its excellent mechanical properties, including flexibility and strength, allow it to withstand physiological loads while maintaining its structural integrity. Additionally, PCL’s low melting point and high thermal stability make it highly processable, enabling precise 3D printing of scaffolds with complex architectures and controlled porosity.^23^ This ensures adequate space for cell migration, vascularization, and nutrient diffusion, which are critical for bone repair. Furthermore, PCL can be easily modified or blended with bioactive agents, such as hydroxyapatite, growth factors, or NPs, to enhance its osteoconductive and osteoinductive properties.^24,25^ PCL-based composites have been widely used to fabricate porous scaffolds that mimic ECM architecture, effectively enhancing osteogenesis in both healthy and osteoporotic bone defects.^26–28^ These advantages make PCL an ideal candidate for fabricating patient-specific scaffolds that promote efficient and effective bone regeneration in orthopedic applications.

Gold plasmonic blackbodies (AuPBs) have emerged as a powerful tool in biomedical applications due to their exceptional photothermal effect, enabling the efficient conversion of near-infrared (NIR) light into localized heat. This property is particularly advantageous for light-activated therapies, such as photothermal therapy (PTT)^29^ and imaging techniques,^30^ including photoacoustic imaging (PAI).^31^ The discovery of the biologically transparent NIR spectral window (at 650–1350 nm) has expanded the applicability of these techniques, particularly in deep-tissue settings.^29^ The second NIR (NIR-II) window (1000–1350 nm) is especially promising, as it offers deeper tissue penetration, reduced background signals, and higher maximum permissible exposure (MPE) compared to the first NIR (NIR-I) window (650–950 nm).^32^ These advantages, combined with the tunable optical properties of AuPBs, enable their use as efficient photothermal transduction agents and imaging probes for precise, targeted diagnostics and therapies. AuPBs incorporated into scaffolds enable precise, laser-induced temperature modulation, creating an optimal thermal microenvironment to support bone regeneration. Moreover, AuPB-loaded scaffolds offer several key advantages. First, their photothermal properties enable noninvasive, on-demand stimulation of bone tissue, potentially reducing reliance on systemic treatments. Second, the homogeneous dispersion of AuPBs within scaffold matrices ensures localized and controlled heating while maintaining the mechanical integrity of the scaffold. Third, the surface properties of AuPBs can be functionalized with bioactive molecules, enhancing their osteoinductive capabilities. Additionally, AuPBs’ strong photothermal activity in the NIR-II window further improves their effectiveness by leveraging the higher MPE and reduced tissue scattering of NIR-II light.

In this study, we developed PCL materials incorporating AuPBs to fabricate filaments for fused deposition modeling (FDM) 3D printing. These filaments were used to print scaffolds designed to evaluate material properties and biological interactions, focusing on the response of MC3T3-E1 preosteoblasts under laser-induced temperature stimulation. In this study, we assessed cell viability and osteogenesis responses to determine the biosafety and osteointegration potential of the scaffolds for bone defect healing in orthopedic applications (Figure 1). Our findings highlight not only the printability and material properties of the AuPB–PCL scaffolds but also their unique capabilities for bone regeneration. (1) Printable filaments for commercial 3D printers: The AuPB–PCL materials were successfully fabricated into 1.75 mm-diameter filaments compatible with widely available 3D printers. (2) Enhanced mechanical properties: Incorporating AuPBs improved the compressive strength of the printed scaffolds, ensuring mechanical stability at the bone defect site. (3) Synergistic photothermal effects: The scaffolds exhibited controlled temperature modulation under NIR laser irradiation, which enhanced osteoblast and osteocyte functions, promoting bone tissue formation. These combined attributes—biocompatibility, mechanical stability, and controllable photothermal stimulation—position the AuPB–PCL scaffolds as a significant advancement in bone tissue engineering, with strong potential for application in orthopedic regenerative medicine.

**Figure 1:**
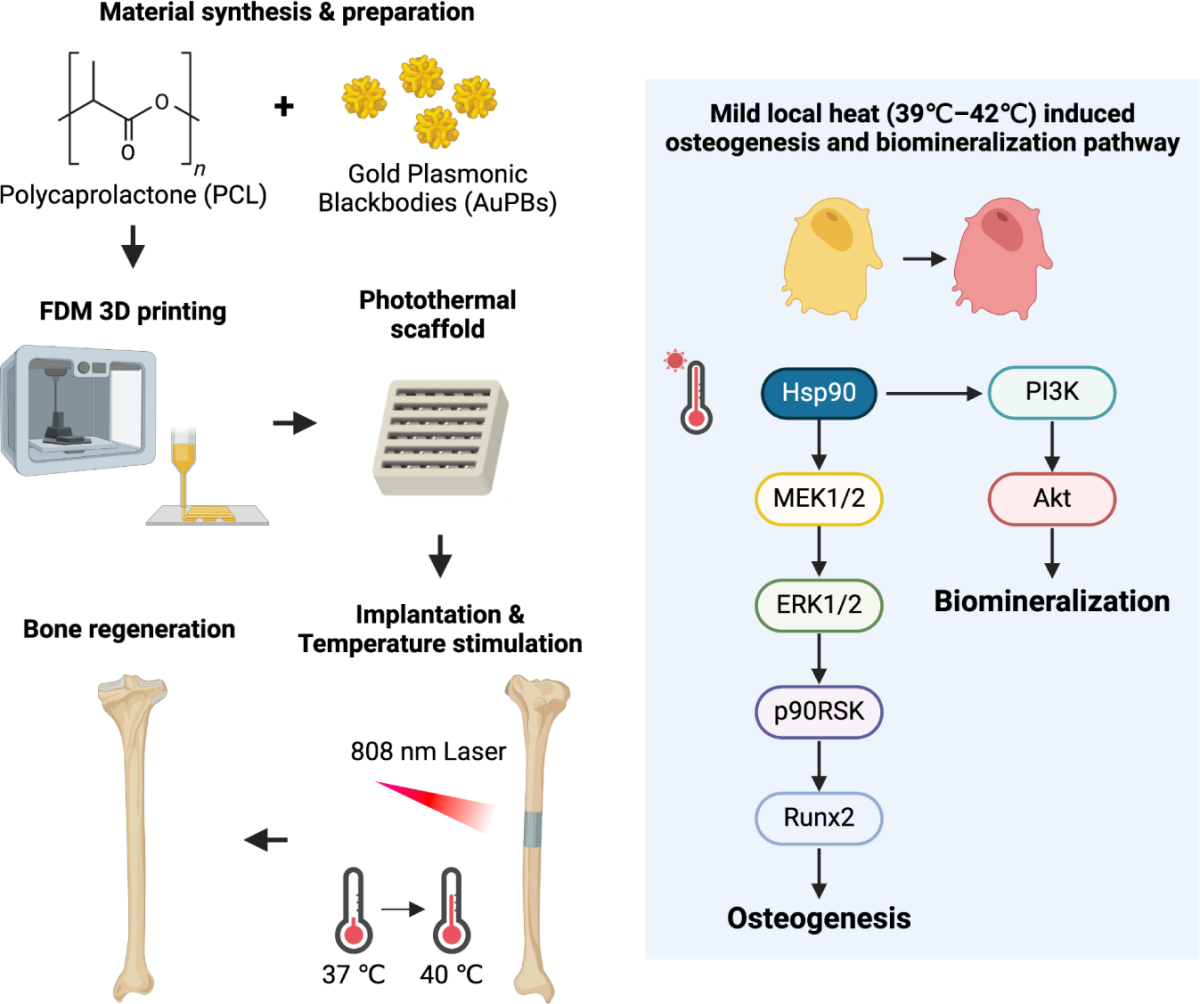
Schematic illustration of the fabrication and application of the photothermal scaffold for bone regeneration. A composite of polycaprolactone (PCL) and gold plasmonic blackbodies (AuPBs) was 3D-printed into a scaffold. After implantation, near-infrared (808 nm) laser irradiation generated mild heat (39–42 °C), which activated cellular signaling pathways that promote osteogenesis and biomineralization, leading to bone regeneration.

## 2 Materials and Experimental Procedures

### 2.1 Study Design

The objective of this study was to develop a biodegradable scaffold with photothermal properties, fabricated via a 3D printing process, as a bone graft for regenerating bone defects. PCL was blended with various concentrations of AuPBs using the melt blending method. Scaffolds were then subjected to 808 nm NIR laser irradiation at a power density of 200 mW/cm^2^, to generate controlled temperature variations for subsequent cell treatments. Filaments were first produced from composite materials and printed under consistent temperature and printing conditions. Comprehensive material characterization and printability assessments of the AuPB–PCL scaffolds were conducted, followed by investigations into the viability and responses of MC3T3-E1 preosteoblasts under elevated temperature conditions. The minimum sample number was *n* = 5 per group.

### 2.2 Composite Filament Preparation

PCL particles (*M*w = 50,000), trade name Capa 6500, were provided by Ingevity (North Charleston, SC, USA). AuPBs were synthesized via a one-pot method. Briefly, 4 mL of a dopamine solution (4 mg/mL) was added to 100 mL of Tris buffer with vigorous stirring. After 5 min, 7 mL of a 0.1 wt % HAuCl_4_ solution was injected into the mixture. The reaction was carried out under continuous stirring for 8 h. The resulting AuPBs were purified by freeze-drying and stored at 4 °C for further use. PCL and AuPB composites were prepared by using the melt blending method. Different concentrations of AuPBs (AuPBs:PCL = 1:100, 2:100, 3:100, 4:100, and 5:100) were first suspended in 75% ethanol and dispersed via ultrasonication. Separately, PCL particles were melted on a hot plate at 100 °C. The AuPB suspension was then added to the molten PCL and stirred for 60 min to ensure a homogeneous dispersion. The mixture was cooled for 24 h and subsequently divided into small pellets, which were extruded at 52 °C and drawn under constant tension to produce 1.75 mm-diameter filaments.

### 2.3 Fabrication of 3D-Printed Composite Scaffolds

The scaffold with dimensions of 8 × 8 × 2.5 mm was printed by using extruded filaments via fused deposition modeling on an E2 3D printer (Raise3D, Shanghai, China) fitted with a 0.4 mm nozzle. The scaffold was constructed using extruded filaments deposited at angles of 0°, 60°, 120°, and 180° in an alternating layered pattern, as shown in the image. The filaments were printed at a temperature of 120 °C, with a deposition velocity of 12 mm/s and a fiber diameter of 330 μm.

### 2.4 Characterization of the AuPB Particle Size and Morphology

The particle size of AuPBs was determined by using dynamic light scattering (DLS) (DKSH, Malvern Instruments, Malvern, UK). The morphology of AuPBs was observed by using transmission electron microscopy (TEM) (HT-7700, Hitachi, Tokyo, Japan), providing high-resolution images of the particles.

### 2.5 Characterization of the Microstructure and Spectral Properties

Samples were fabricated into plate shapes with a diameter of 10 mm and a thickness of 0.5 mm by using a mold. The absorption spectra of the samples were analyzed using an ultraviolet (UV)–visible (vis)–NIR spectrophotometer (EE2063-050-FUVN model, OtO Photonics, Hsinchu, Taiwan) over a scan range of 300–1100 nm. Fourier transform infrared (FTIR) spectroscopy (Nicolet Summit Pro, Thermo Fisher Scientific, Pittsburgh, PA, USA) was used to observe and characterize the compositions of PCL samples that incorporated various amounts of AuPBs. The thermal properties of the samples were evaluated by using differential scanning calorimetry (DSC) (DSC404 F3, Netzsch, Selb, Germany) to determine the melting temperature after AuPB incorporation.

### 2.6 Evaluation of Surface Wettability

The surface wettability of the samples was evaluated by measuring the water contact angle. Samples were first washed with 99.5% ethanol and dried in an oven. Deionized (DI) water droplets were deposited on the sample surface by using the sessile drop method. Images of the droplets and sample surfaces were captured with a camera, and contact angles were analyzed using ImageJ software (National Institutes of Health, Bethesda, MD, USA).

### 2.7 Temperature Response under Laser Irradiation

The relationship between the AuPB concentration in PCL scaffolds and the temperature change induced by laser irradiation was analyzed to determine the optimal working temperature range for subsequent cell culture experiments. Scaffolds were placed in a 24-well culture plate, and an 808 nm laser was focused onto the bottom of the plate, passing through the plate to reach the scaffold. This setup ensured uniform exposure to the laser while minimizing potential external interference. Prior to irradiation, the laser power was measured using a power meter and adjusted to 200 mW/cm^2^. The temperature change in the scaffold under laser irradiation was monitored and recorded using a thermocouple and a thermal imaging camera at specific time intervals: 10 s, 20 s, 30 s, 40 s, 50 s, 1 min, 2 min, 3 min, 4 min, 5 min, 10 min, 30 min, and 60 min. These measurements were conducted to evaluate the temperature profile over time.

### 2.8 Compression Test

Compressive strength of the scaffolds was determined by a compression test using a universal testing system (Pin Tai Technology, Taichung, Taiwan). Scaffold samples with dimensions of 8 × 8 × 2.5 mm were compressed at a crosshead speed of 6 mm/min until a strain of 20%, and the corresponding stress was recorded as the compressive strength.

### 2.9 Printability Test

The printability of scaffolds fabricated with PCL containing different AuPB contents was evaluated using scanning electron microscopy (SEM) (SU-7700, Hitachi, Tokyo, Japan) and microcomputed tomography (micro-CT) (SKYSCAN 1176; Bruker, Billerica, MA, USA). After printing, scaffolds were sectioned to observe the cross-sectional and top-surface morphologies using SEM. Additionally, scaffolds were scanned with a micro-CT system, and the acquired data were reconstructed into 3D models using commercial software. Structural properties, including strut thickness, strut area, pore size, and pore volume, were analyzed to compare the scaffolds with various AuPB contents.

### 2.10 Cell Viability Test

The biocompatibility of scaffolds with various AuPB contents was assessed using 3-(4,5-dimethylthiazol-2-yl)-2,5-diphenyltetrazolium bromide (MTT) and Live/Dead assays in MC3T3-E1 preosteoblasts. Scaffolds were placed into a 48-well culture plate, followed by the addition of a cell suspension at a density of 3000 cells/well. Cells were incubated for 24 h at 37 °C in a 5% CO_2_ atmosphere. On days 1, 3, and 5, scaffolds were irradiated with an 808 nm NIR laser for 10 min to generate controlled photothermal heating. On day 7, 10 μL of an MTT solution (Invitrogen, Carlsbad, CA, USA) was added to each well and incubated for 3 h. Subsequently, 100 μL of dimethyl sulfoxide (DMSO) was added to dissolve the formazan crystals, and the optical density (OD) was measured at 560 nm using an enzyme-linked immunosorbent assay (ELISA) reader (Multiskan FC; Thermo Fisher Scientific, Waltham, MA, USA).

### 2.11 Cell Morphology Observation

The cell morphology of plate samples (10 mm in diameter and 1 mm in thickness) with various AuPB contents was evaluated using crystal violet staining of MC3T3-E1 preosteoblasts. Plate samples were placed in a 24-well culture plate, and a cell suspension at a density of 5000 cells/well was added. Cells were incubated for 24 h at 37 °C in a 5% CO_2_ atmosphere. On days 1, 3, and 5, the scaffolds were irradiated with an 808 nm NIR laser for 10 min to generate various temperatures. On day 7, cells were washed with phosphate-buffered saline (PBS), fixed with 4% paraformaldehyde, and subsequently stained with crystal violet dye. Stained cells were visualized and imaged on an upright microscope (BX53, Olympus, Tokyo, Japan). ImageJ software was used to evaluate the cell aspect ratio for measuring and analyzing cell morphologies.

### 2.12 Osteogenesis Response Staining and Analysis

Plate samples (10 mm in diameter and 1 mm in thickness) containing various AuPB contents were evaluated by using crystal violet staining of MC3T3-E1 preosteoblasts. Plate samples were placed in a 24-well culture plate, and a cell suspension at a density of 5000 cells/well was added. Cells were incubated at 37 °C in a 5% CO_2_ atmosphere for 24 h to allow cell attachment. On days 1, 3, 5, 7, 11, 13, 15, 17, and 19, scaffolds were irradiated with an 808 nm NIR laser for 10 min to generate controlled photothermal heating. On day 14, cells were washed with PBS and fixed with 4% paraformaldehyde. Osteogenesis was assessed using ALP alizarin red S (ARS), and collagen-1 staining was used to evaluate osteogenesis signals and calcium deposition. Images were first captured with an optical microscope (CKX53; Olympus, Tokyo, Japan). The area stained for ALP, ARS, and collagen-1 was quantified by using ImageJ software.

### 2.13 Statistical Analysis

All results in this study are expressed as the mean ± standard deviation (SD). Statistical analyses were conducted using SPSS software (version 20.0, IBM, Armonk, NY, USA). Data were analyzed through a *t*-test and one-way analysis of variance (ANOVA), followed by Tukey’s post-hoc test. A *p*-value of <0.05 was considered statistically significant.

## 3 Results and Discussion

### 3.1 Characterization of AuPBs

The synthesized AuPBs exhibited a particle size of approximately 100 nm, as confirmed by both DLS and TEM analyses (Figure 2a). DLS measurements provided an average hydrodynamic diameter, while TEM images offered direct visualization of the NPs, confirming their uniform size and spherical morphology. The close agreement between DLS and TEM results indicates a narrow size distribution, suggesting that the synthesis process successfully yielded monodispersed AuPBs.

**Figure 2:**
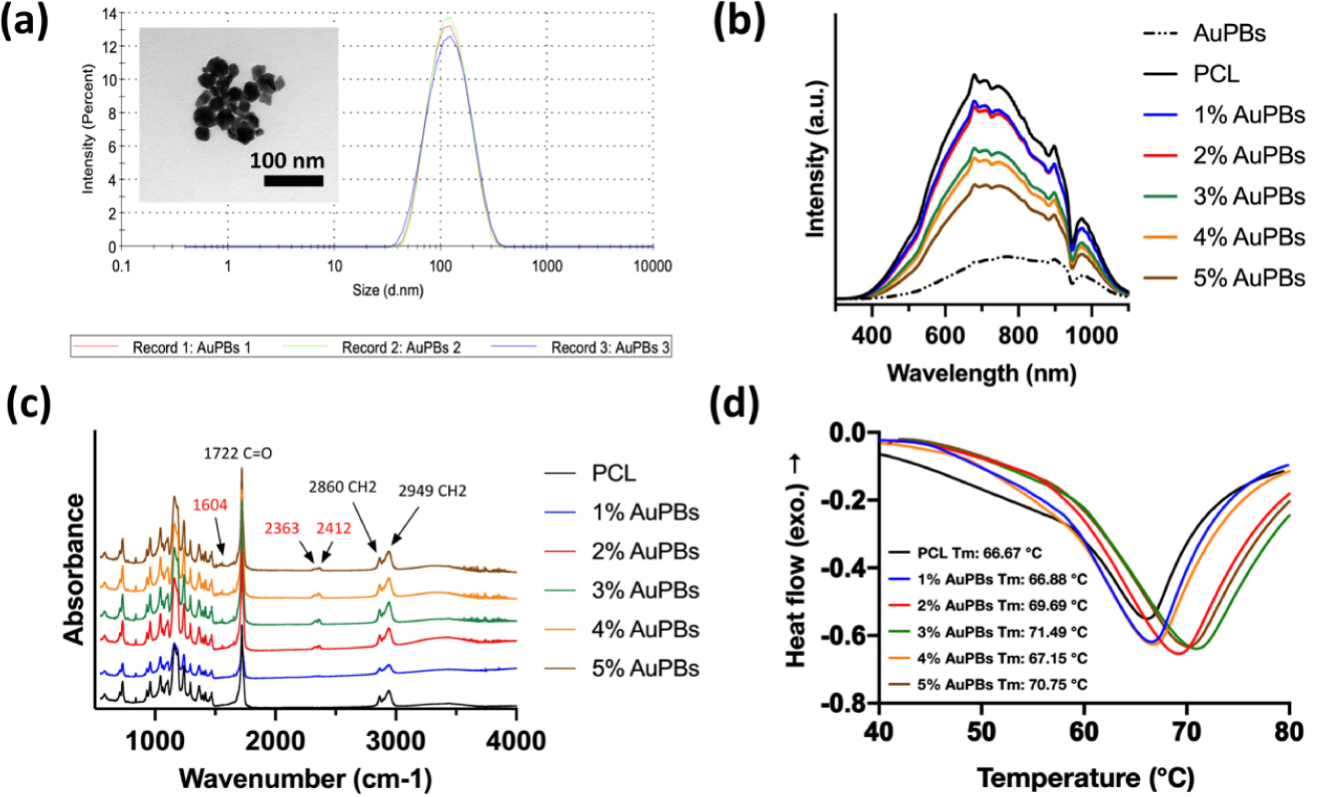
Comprehensive characterization of gold plasmonic blackbodies (AuPBs) and AuPB–polycaprolactone (PCL) scaffolds: (a) particle size distribution and TEM image of AuPBs, (b) UV–vis–NIR absorption spectrum of AuPB–PCL materials, (c) FTIR spectrum of AuPB–PCL materials, and (d) thermal properties of AuPB–PCL materials.

The uniform size distribution and monodispersity of NPs are crucial for localized surface plasmon resonance (LSPR) and significantly impact photothermal conversion efficiency.^33^ The photothermal efficiency decreases as the size of AuNPs increases.^34^ The uniform dispersion of these monodispersed AuPBs within the PCL matrix ensures a homogeneous temperature distribution during photothermal treatment, minimizing the risk of localized overheating that could negatively impact surrounding cells.^35^ Nanomaterials with a size of approximately 100 nm are particularly suitable for incorporation into matrix materials, as they tend to disperse uniformly and homogeneously. Additionally, NPs of this size are considered optimal for endocytosis, potentially facilitating cellular uptake and metabolism, which may further contribute to bone regeneration.^36^

### 3.2 Absorption Spectrum of PCL Composite Materials

Figure 2b presents the UV–vis–NIR absorption spectra of the PCL-AuPB composite materials. The incorporation of AuPBs into the PCL matrix resulted in a significant increase in absorption intensity across the 600–1000 nm range, indicating successful NP integration. The absorption spectrum showed a broad plasmonic resonance, which is characteristic of AuPBs, and its intensity progressively increased with higher AuPB concentrations. The most notable absorption enhancement occurred in the 1–3% AuPB groups, suggesting optimal dispersion of NPs within the PCL matrix. However, at 4% and 5% AuPBs, the absorption increases were less pronounced, potentially due to particle aggregation affecting optical interactions.

A key factor influencing the photothermal efficiency of the composite material is the localized SPR (LSPR) effect of nanomaterials.^37^ The observed increase in absorption intensity suggests that AuPBs effectively absorbed light in the NIR region, which is critical for applications such as photothermal therapy (PTT) and bone tissue engineering.^29^ Since the photothermal conversion efficiency depends on the size, shape, and concentration of NPs, the results imply that an optimized AuPB concentration can enhance light absorption and heat generation within the scaffold, contributing to controlled thermal stimulation for biomedical applications.

### 3.3 Characterization of PCL Composite Materials

Figure 2c shows the FTIR spectra of PCL composite materials, highlighting characteristic PCL peaks at 1722, 2860, and 2949 cm^–1^, corresponding to vibrations of the polymer backbone. These are attributed to C=O stretching and symmetric/asymmetric C–H stretching, respectively.^38^ Additional peaks at 1604, 2363, and 2412 cm^–1^ were observed, further confirming the presence of AuPBs within the composite matrix. These new peaks may originate from surface functional groups on the AuPBs or carbon-based species adsorbed during synthesis, such as C=C aromatic ring stretching or CO_2_-related vibrations.^39^ The coexistence of these characteristic peaks in the FTIR spectra verifies the successful incorporation of AuPBs into the PCL matrix. Furthermore, retention of PCL-specific peaks indicates that the polymer structure remained intact following the addition of AuPBs, while the appearance of AuPB-specific peaks suggests effective dispersion and interaction with the polymer.

Characteristic peaks of PCL at 1722 cm^–1^ (C=O stretching) and 2860 and 2949 cm^–1^ (C–H stretching) remained prominent, suggesting that the fundamental polymer backbone remained unchanged upon AuPB addition. This retention is crucial, as it ensures that the mechanical and degradative properties of PCL were not significantly compromised by NP incorporation. The presence of new peaks at 1604, 2363, and 2412 cm^–1^ further supports the occurrence of molecular-level interactions between AuPBs and the PCL matrix. These peaks could be attributed to surface functional groups on the AuPBs or potential chemical interactions between the polymer and NPs. The presence of these peaks suggests that AuPBs were well integrated and dispersed within the composite rather than merely forming aggregates or phase-separated domains. Such nanoscale interactions may contribute to enhanced interfacial bonding, resulting in improved thermal, mechanical, and optical properties of the material.

### 3.4 Thermal Properties of PCL Composite Materials

DSC profiles of PCL samples incorporating various AuPB contents are shown in Figure 2d. The melting temperature of the pure PCL sample was observed to be 66.67 °C. Upon incorporation of 3% AuPBs, the melting temperature increased to 71.49 °C. This shift suggests that the addition of AuPBs enhanced the thermal stability of the PCL matrix. These results demonstrate that incorporating AuPBs not only modified the thermal behavior of PCL but also opened possibilities for tailoring its properties for applications requiring enhanced thermal stability.

The incorporation of NPs within the PCL matrix can induce physical and chemical interactions, such as hydrogen bonding and van der Waals forces, which enhance material cohesion and contribute to improved thermal stability.^40^ Additionally, NPs can act as reinforcement centers, restricting the mobility of polymer chains under thermal stress and thereby delaying the melting process. This phenomenon improves mechanical stability and thermal resistance, making the material more durable under physiological conditions.^41^

### 3.5 Water Contact Angle of PCL Composite Samples

Figure 3a presents water contact angle measurements of the PCL composite samples. Following AuPB incorporation into the PCL matrix, the contact angle remained approximately 70°, with no significant differences observed among the groups. This stability in the water contact angle suggests that the inclusion of AuPBs did not markedly influence the surface wettability of the composites, implying that the AuPBs were likely well-integrated into the PCL matrix without significant alteration of the surface chemistry or topography. Incorporating AuPBs did not significantly alter the surface microtopography or wettability based on the contact angle measurements, resulting in minimal surface energy modification, which is a key factor in determining their hydrophilic or hydrophobic properties.^42^

**Figure 3:**
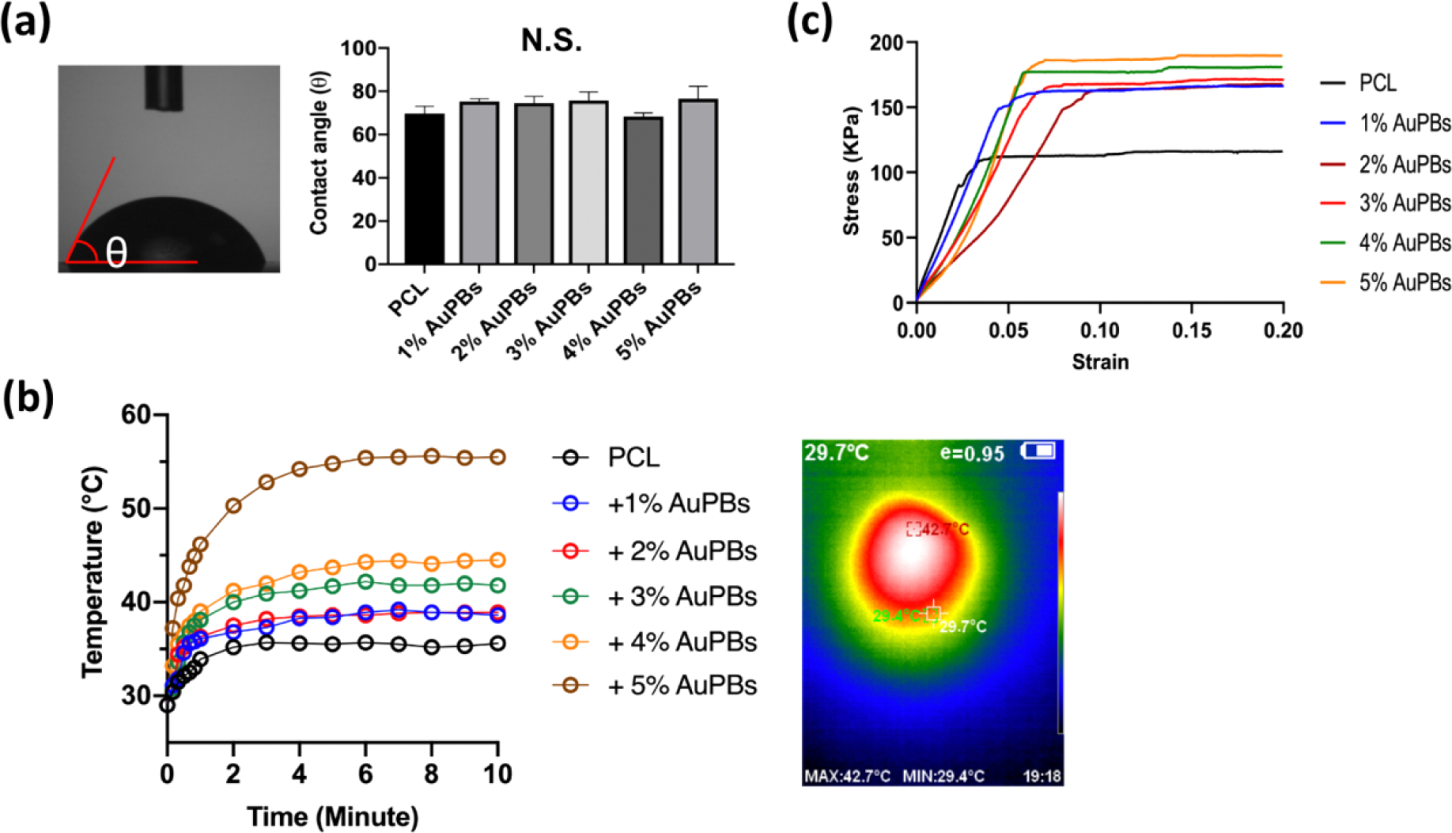
Additional characterization of gold plasmonic blackbody (AuPB)–polycaprolactone (PCL) scaffolds. (a) Water contact angle measurement of AuPB–PCL plates, (b) temperature variations of AuPB–PCL scaffolds under 808 nm laser irradiation at a power density of 200 mW/cm^2^, and (c) compression strength of AuPB–PCL scaffolds.

### 3.6 Temperature Variations of PCL Composite Scaffolds

Temperature variations of PCL samples incorporating various AuPBs under irradiation by an 808 nm NIR laser are shown in Figure 3b. The temperature in all groups rapidly rose during the initial 4 min of irradiation, after which it stabilized, making it suitable for subsequent *in vitro* studies. The final temperatures achieved were 35.6 °C for pure PCL and 38.6, 38.9, 41.8, 44.5, and 55.5 °C for PCL composites containing 1%, 2%, 3%, 4%, and 5% AuPBs, respectively. This trend indicates that the inclusion of AuPBs significantly enhanced the photothermal conversion efficiency of the composites, with higher AuPB contents resulting in greater temperature increases. The rapid heating followed by stabilization demonstrates the capability of these materials to achieve and maintain controlled hyperthermic conditions, which is essential for applications such as PTT or heat-assisted tissue engineering.

For PTT, elevated temperatures above 42 °C are commonly utilized for cancer treatment, thrombolysis management, and antibacterial applications.^43,44^ However, moderate hyperthermic conditions (39–42 °C) were shown to promote osteogenesis and bone cell proliferation, making them particularly relevant for bone regeneration strategies. This effect is achieved by dynamically regulating the inducible nitric oxide synthase/arginase 1 (iNOS/Arg1) balance, which plays a crucial role in modulating inflammatory responses and tissue remodeling. By maintaining this equilibrium, controlled hyperthermia can enhance osteogenic differentiation while minimizing excessive inflammatory activation.^45,46^ In this context, PCL composites containing 2–3% AuPBs, which stabilized within this temperature range, offer promising potential for biomedical applications. The observed temperature stabilization after the initial heating phase suggests a balance between heat generation and dissipation, which is essential for ensuring safe and controlled hyperthermia-based therapies. This controlled temperature plateau is particularly significant in biomedical settings, as it helps prevent excessive heating, which could otherwise lead to unintended cell damage or protein denaturation.^47^

### 3.7 Mechanical Properties of PCL Composites Scaffolds

The stress–strain curve of PCL composite printed scaffolds is shown in Figure 3c. The maximum compressive stress of the pure PCL scaffold was approximately 120 kPa. However, the compressive stress significantly increased after incorporating AuPBs, reaching values of 160–180 kPa. Notably, scaffolds with higher AuPB contents exhibited greater compressive strengths, indicating a concentration-dependent enhancement of mechanical performance. This improvement in mechanical properties can be attributed to the reinforcing effect of AuPBs, which likely enhanced the load-bearing capacity of the PCL matrix by improving its structural integrity and resistance to deformation under compressive forces. The uniform distribution of AuPBs within the PCL matrix may have contributed to effective stress transfer between the polymer and the NPs. These results suggest that incorporating AuPBs into PCL scaffolds can enhance their mechanical performance, making them more suitable for applications requiring greater mechanical stability, such as bone tissue engineering.

This improvement aligns with previous studies on NP-reinforced polymer scaffolds, where the inclusion of rigid nanomaterials effectively restricted polymer chain mobility, leading to enhanced mechanical properties and structural integrity.^48^ Similarly, in our study, the compressive strength increased with higher AuPB concentrations, suggesting that AuPBs serve as effective load-bearing reinforcements, improving stress distribution and deformation resistance, thereby further enhancing the mechanical stability of the material. Despite these improvements, the compressive strength and modulus of AuPB–PCL scaffolds remained lower than those of human cancellous bone, which typically exhibits a compressive modulus of 75–435 MPa and a compressive strength of 2–12 MPa.^49^ Notably, incorporating 5% AuPBs resulted in an approximate 50% increase in the compressive strength compared to pure PCL scaffolds. However, our results indicated that the mechanical performance of pure PCL scaffolds in this study was significantly lower than previously reported values,^50^ suggesting that the scaffold architecture plays a crucial role in the mechanical behavior. Maintaining mechanical compatibility with the host bone is essential for scaffold integration, stability, and long-term functionality. Additionally, an adequate load-bearing capacity ensures that the scaffold can withstand physiological hydrostatic and pulsatile pressures while preserving porosity for cell attachment, proliferation, and differentiation.^51,52^ Further optimization of the scaffold design and fabrication parameters could be explored to enhance mechanical performance while maintaining biofunctionality for bone tissue engineering applications.

### 3.8 Printability of PCL Scaffolds with Various AuPB Concentrations

Figure 4 illustrates the surface topography of the prepared filaments and printed scaffolds using PCL materials incorporating various AuPB contents. SEM images of the top view of the prepared filaments reveal smooth surfaces with scratch marks caused by the nozzle during filament preparation. Cross sections of the filaments exhibit flat surfaces, with melting traces observed during the cutting process. In contrast, the top view of the printed scaffolds prepared with pure PCL showed smooth surfaces. However, with AuPB incorporation, the printed surfaces began to exhibit protrusions. Higher AuPB contents resulted in more pronounced protrusions. Specifically, scaffolds incorporating 2% and 3% AuPBs displayed vein-like and nonuniform patterns on their surfaces. Furthermore, cross sections of the scaffolds exhibited nonuniform patterns across different groups, suggesting the presence of internal stress concentrations caused by AuPB incorporation.

**Figure 4:**
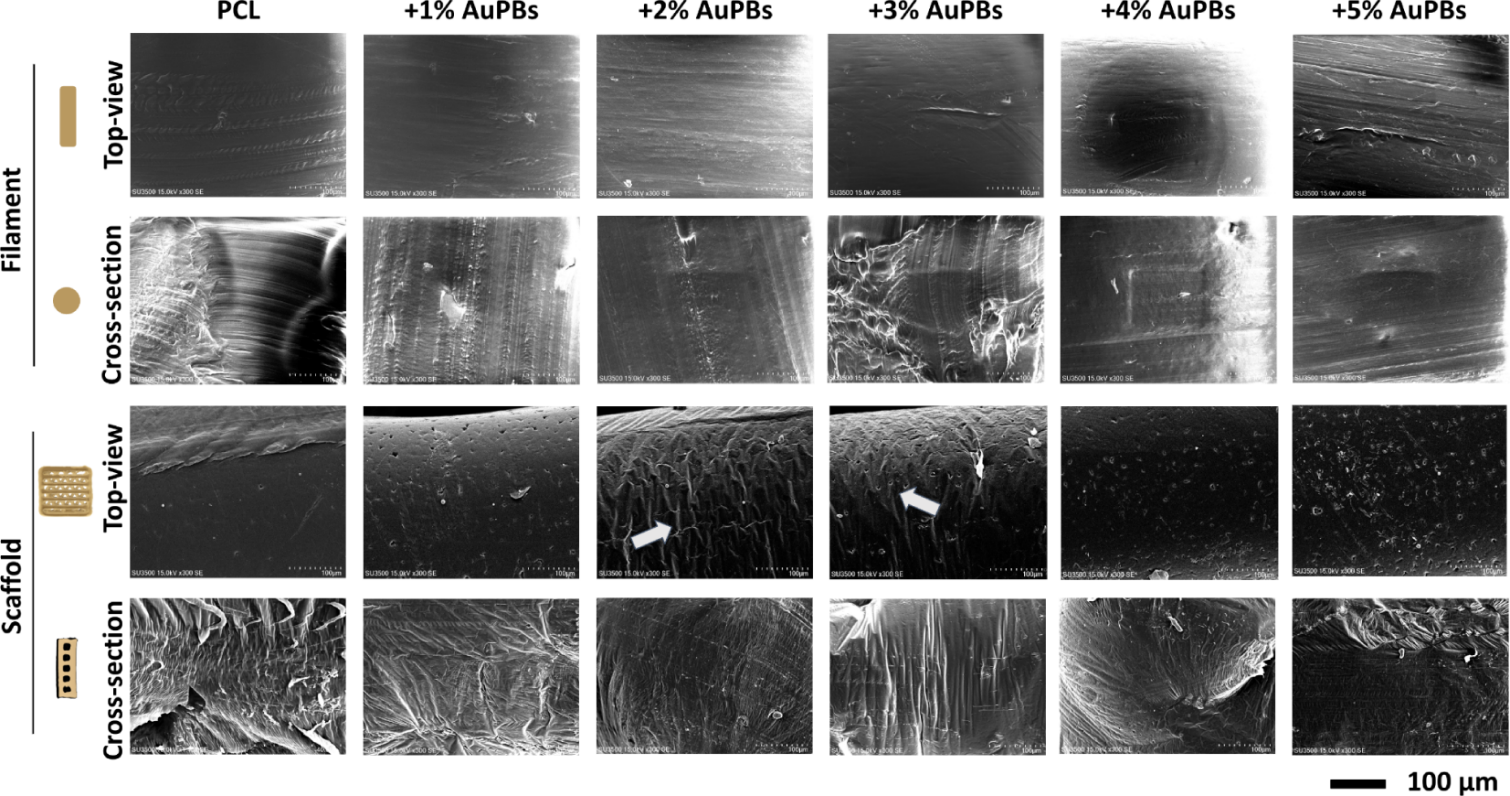
SEM observation of the surface morphology of printed gold plasmonic blackbody (AuPB)–polycaprolactone (PCL) scaffolds with various AuPB contents.

Data from the CT scan were reconstructed to generate a 3D model. Figure 5a displays a macro view of the printed scaffold models prepared using PCL materials with various AuPB contents. The deposited angles of the extruded filaments showed no notable differences among the groups. Detailed visualization of scaffold structures is presented in Figure 5b, while quantitative results are shown in Figure 5c. The horizontal strut thickness in the first layer of the scaffolds demonstrated variations in the printed thickness across different groups, with significant differences observed compared to those in the designed model. The diagonal strut area in the second layer revealed different pattern types; however, no significant differences were found compared with the designed model. When the pore size was visualized from a side view, the pore topography was clearly observed but still differed from the designed model. Finally, the volumes of the scaffolds across different groups were measured, and no significant differences were detected among the groups. In conclusion, incorporating various AuPB contents into PCL materials not only influenced the properties of the prepared filaments but also had a measurable impact on the quality of the printed scaffolds.

**Figure 5:**
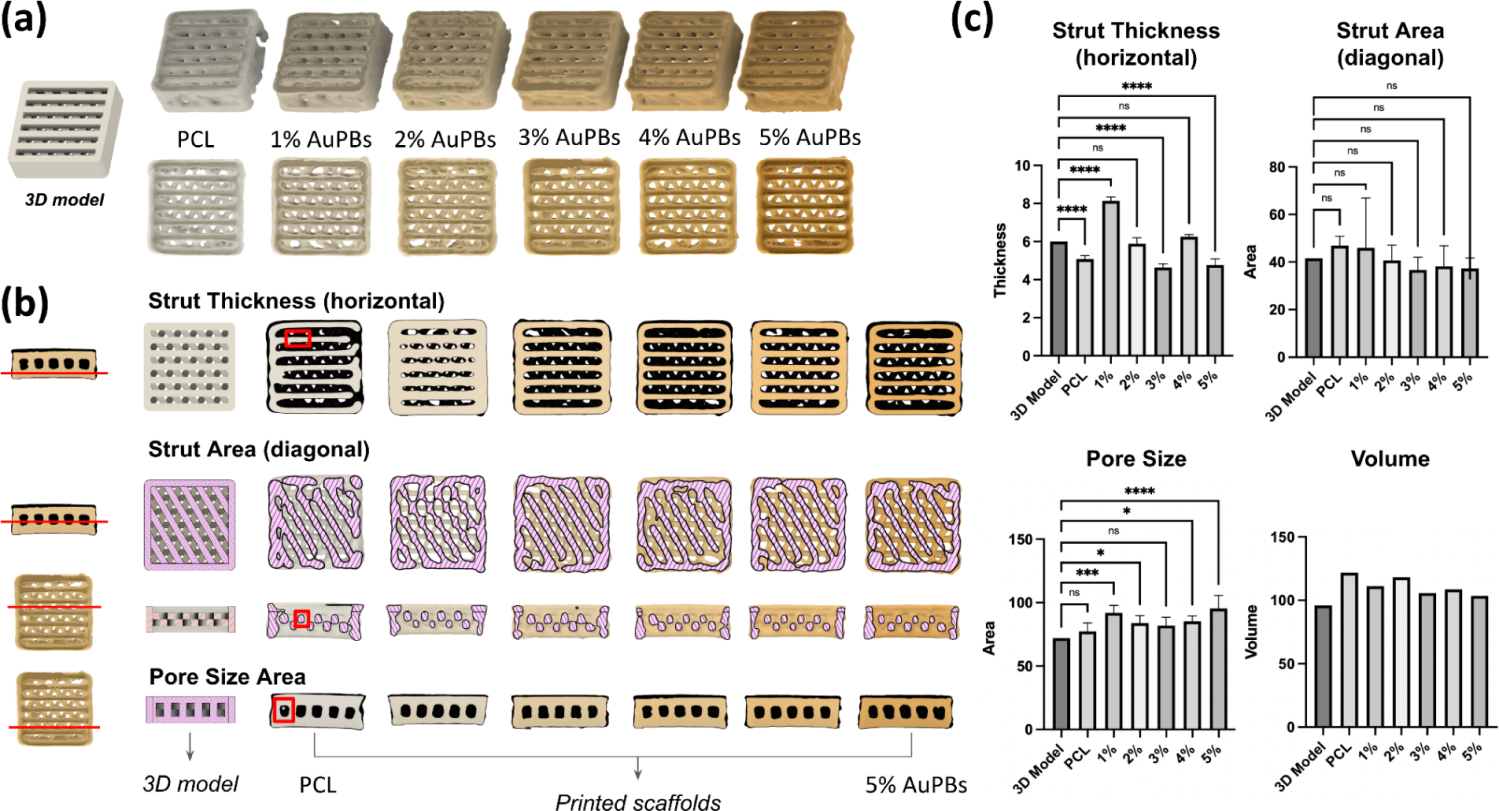
Effects of various gold plasmonic blackbody (AuPB) amounts on the postprinting surface morphology of scaffolds analyzed using micro-CT. (a) Photographs of printed scaffolds with different concentrations of AuPBs (0–5%), compared to the original 3D model. (b) Cross-sectional micro-CT images used to analyze morphological parameters, including horizontal strut thickness, diagonal strut area, pore size area, and total volume. Colored overlays highlight the analyzed regions. (c) Quantitative analysis of strut thickness, strut area, pore size, and scaffold volume (*n* = 5 per group; *p < 0.05, ***p < 0.005, and ****p < 0.001; N.S., not significant).

Incorporating AuPBs into PCL scaffolds induced significant modifications in the surface morphology, internal structure, and dimensional accuracy, indicating that the nanomaterials influenced the extrusion dynamics, flow behavior, and solidification process of the composite material.^53^ The presence of nonuniform patterns in the scaffold cross sections suggested the formation of internal stresses during the printing process, likely resulting from differences in the thermal expansion coefficients between PCL and AuPBs.^54^ These findings highlight the need for further optimization of material processing and printing parameters to enhance the structural uniformity and mechanical stability of AuPB–PCL scaffolds.

### 3.9 Viability and Morphology of MC3T3-E1 Preosteoblasts Cultured on PCL Scaffolds with Various AuPB Concentrations at Different Temperatures

The viability and morphology of MC3T3-E1 preosteoblasts were evaluated to assess the biocompatibility of and the cellular response to PCL scaffolds containing various concentrations of AuPBs under different temperature conditions. Laser irradiation was applied to induce a photothermal effect, creating a localized thermal microenvironment on the scaffolds. The results, shown in Figure 6a,b, demonstrate that cell viability varied based on both the concentration of AuPBs and the application of laser irradiation. Scaffolds irradiated with an NIR laser generally exhibited higher cell viability compared to nonirradiated groups, highlighting the beneficial effects of photothermal stimulation. However, it is noteworthy that excessive heat generation, particularly at higher AuPB concentrations combined with laser irradiation, may lead to thermal stress that negatively impacts cell viability. This suggests that there is an optimal thermal window for photothermal stimulation—mild hyperthermia can promote cellular activity, while temperatures exceeding this range may induce cell damage or apoptosis. These findings underscore the importance of controlling both the AuPB concentration and irradiation conditions to maintain a favorable thermal microenvironment for cell survival and function. While laser irradiation significantly enhanced cell viability compared to the PCL group without AuPBs, no significant differences were observed between the irradiated and nonirradiated groups of the same material (Figure 6b). Enhanced cellular growth was observed in the irradiated groups, except for plates with the highest AuPB incorporation, which generated excessive temperatures. These findings emphasize the potential of AuPB-loaded PCL scaffolds to foster a favorable environment for osteoblast proliferation and activity under photothermal conditions, provided the temperature is carefully controlled.

**Figure 6:**
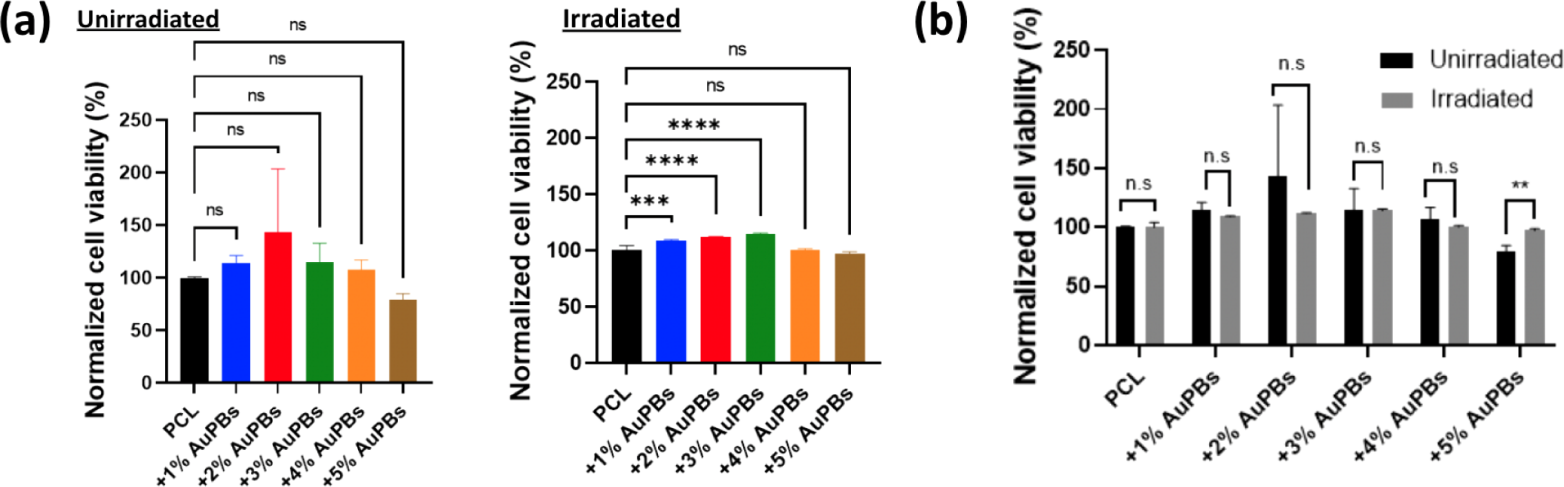
Viability of MC3T3-E1 preosteoblasts cultured on polycaprolactone (PCL) scaffolds with various gold plasmonic blackbody (AuPB) concentrations at different temperatures. (a) Cell viability across different material groups with and without laser irradiation, and (b) comparative analysis of cell viabilities for the same material with and without laser irradiation (*n* = 5 per group; ***p < 0.005 and ****p < 0.001; N.S., not significant).

Crystal violet staining (Figure 7a) provided qualitative insights into cell morphology, showing uniform cell attachment and spreading across scaffold surfaces. A quantitative analysis of the cell aspect ratio, which reflects the elongation and shape of cells, is presented in Figure 7b,c. The aspect ratio is a key morphological parameter used to assess cellular responses to biomaterials, as changes in cell shape can indicate variations in adhesion, cytoskeletal organization, and mechanotransduction processes. In Figure 7b, the unirradiated group exhibited no significant differences in aspect ratios among the different material groups, suggesting that AuPB incorporation alone, without laser activation, did not influence cell elongation under normal culture conditions. However, in the irradiated group, cells cultured on PCL plates incorporating 2%, 3%, and 4% AuPBs exhibited higher aspect ratios, indicating that photothermal stimulation influenced cellular morphology. Notably, the 3% AuPB group showed a statistically significant increase, suggesting that this specific concentration may have provided an optimal photothermal effect that enhanced cytoskeletal remodeling and cell elongation. Further comparisons between irradiated and unirradiated conditions for the same material (Figure 7c) revealed that only the 3% AuPB group exhibited a significant difference in the aspect ratio. This finding suggests that moderate photothermal stimulation (achieved with 3% AuPBs) may enhance cytoskeletal reorganization, promoting cell elongation and potentially facilitating osteogenic differentiation. In contrast, lower AuPB concentrations might not generate sufficient thermal stimulation, while higher concentrations (e.g., 4% AuPBs) may induce excessive heat, potentially leading to stress responses that limit cellular elongation.

**Figure 7:**
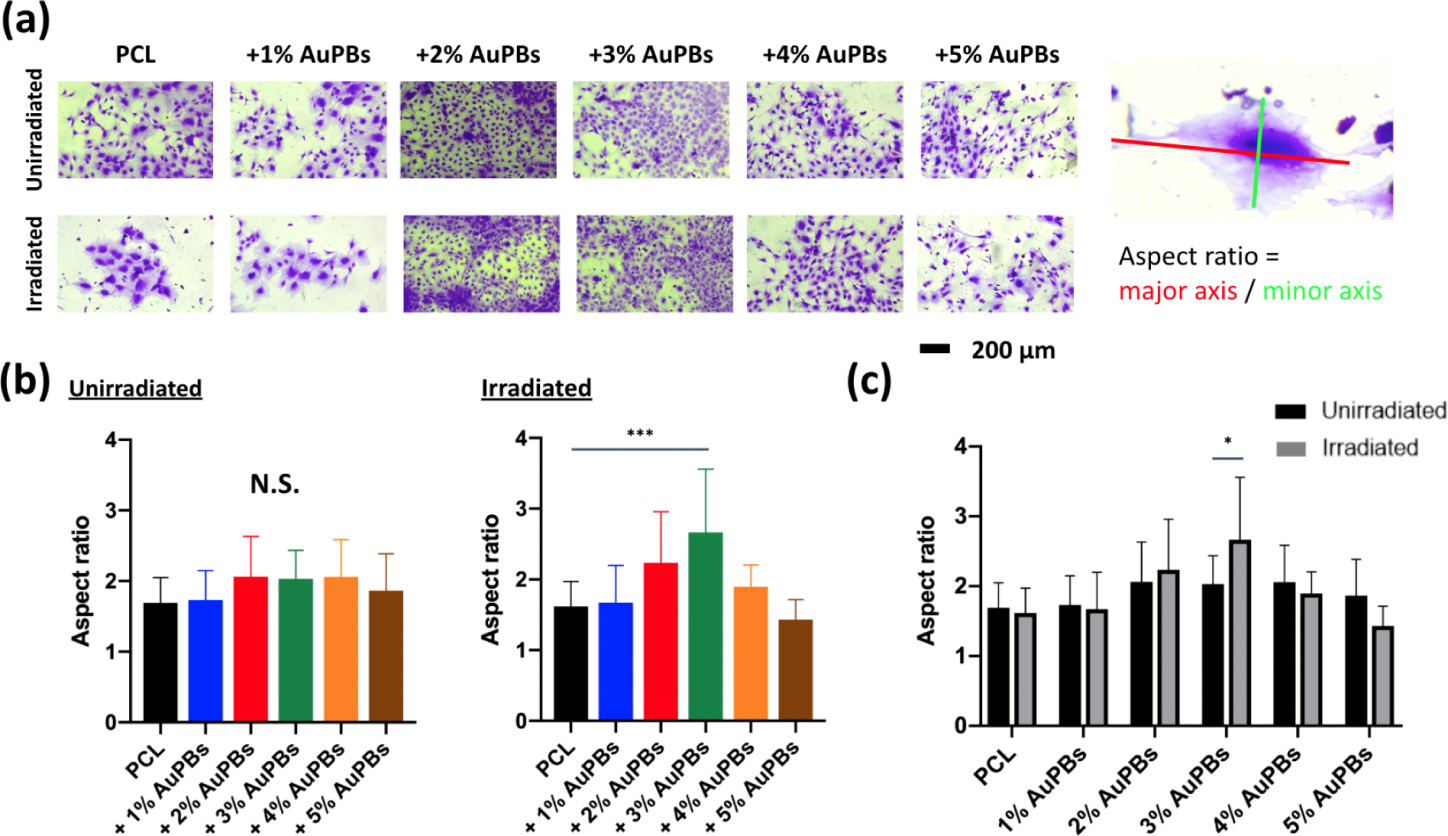
Cell morphology of MC3T3-E1 preosteoblasts cultured on a polycaprolactone (PCL) plate with various gold plasmonic blackbody (AuPB) concentrations at different temperatures. (a) Representative images of cell morphology were visualized using crystal violet staining. (b) The aspect ratio of cells across different material groups with and without laser irradiation. (c) Comparative analysis of the aspect ratios of cells for the same material with and without laser irradiation (*n* = 10 per group; *p < 0.05 and ***p < 0.005; N.S., not significant).

Gold nanomaterials exhibit excellent biocompatibility, low cytotoxicity, and strong oxidative resistance, with their size, shape, and functionalization enabling diverse biomedical applications such as drug delivery,^55^ diagnostic imaging,^56^ and targeted therapy.^57,58^ Their ability to traverse biological barriers and influence cell proliferation and differentiation has been widely studied. However, reports on their cytotoxicity remain conflicting, as their biological effects are size-, concentration-, and time-dependent.^59,60^ Our findings confirmed that AuPBs (∼100 nm) promoted cell proliferation, supporting their potential for tissue engineering applications. Laser irradiation in combination with gold nanomaterials can induce localized temperature increases, stimulating bone formation.^10^ Previous studies demonstrated that mild hyperthermia (39–41 °C) promotes osteoprogenitor proliferation and differentiation, whereas higher temperatures (≥42.5 °C) or prolonged exposure (>96 h) inhibit proliferation. Notably, short-term heat shock (1 h at 42.5 °C) increased ALP expression, while continuous exposure to 39 °C enhanced mineralization. The upregulation of HSP70 at 39 °C suggested its role in osteogenic signaling and cellular protection, further supporting the potential of controlled photothermal stimulation from AuPB–PCL scaffolds for bone regeneration within an optimal temperature range. Additionally, elevated temperatures were shown to significantly inhibit the proliferation of osteosarcoma-derived cell lines, including osteosarcoma-derived cell lines HOS85, MG-63, and SaOS-2. A sublethal heat shock (42 °C, 1 h) was reported to decrease ALP activity, indicating that thermal exposure may have differential effects on normal and cancerous bone cells.^61^ These findings highlight the importance of precise thermal regulation in photothermal-based bone regeneration therapies, ensuring enhanced osteogenic activity while minimizing potential cytotoxic effects.

The aspect ratio (AR) is a key parameter in cell shape regulation, influencing cellular behavior and differentiation. The Mrksich group reported a monotonic increase in osteogenic differentiation with an increasing AR,^62^ whereas Ding et al. found a nonmonotonic response, identifying an optimal AR of ∼2 for osteogenesis.^63^ These findings align well with our results, demonstrating a correlation between AR and osteogenesis.

### 3.10 Osteogenesis and Mineralization Responses of MC3T3-E1 Preosteoblasts on AuPB–PCL Scaffolds with Various AuPB Contents at Different Temperatures

ALP is a key bone matrix protein that plays a vital role in bone formation and mineralization. As a widely recognized marker of osteoblast differentiation, ALP activity is closely associated with bone matrix synthesis, making it an essential indicator for evaluating osteogenic potential. Figure 8a displays purple-stained regions representing ALP expression across different groups. Quantification using ImageJ software revealed that ALP activity was consistently higher in irradiated groups incorporating AuPBs in PCL scaffolds compared to nonirradiated groups. Among them, the 5% AuPB group exhibited the highest ALP expression, with a normalized stained area reaching 1716% relative to the nonirradiated control group. This substantial increase suggests that photothermal stimulation, particularly at higher AuPB concentrations, enhanced osteoblast function by promoting ALP production, likely due to a higher localized temperature and improved cellular activity.^64^

**Figure 8:**
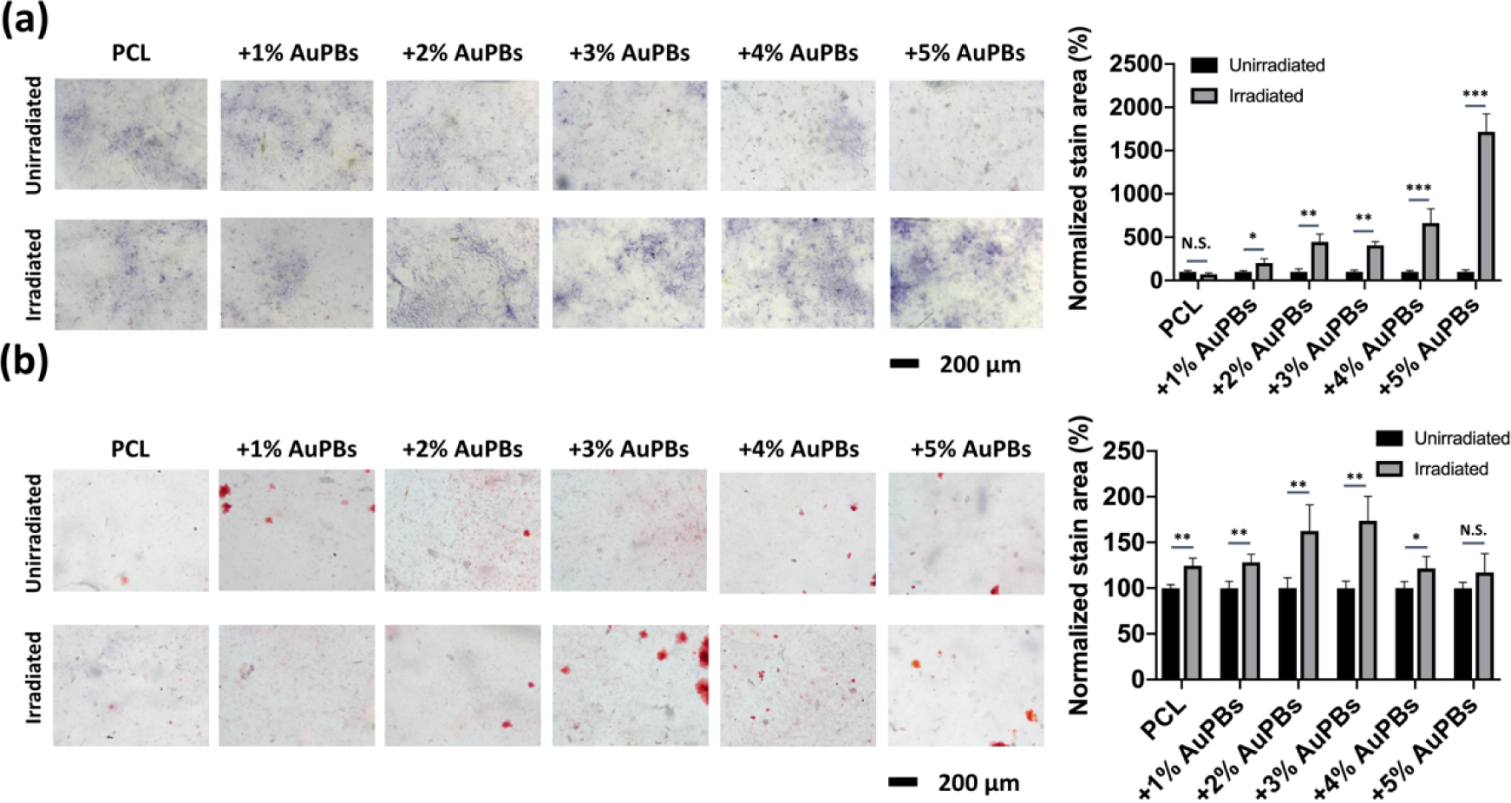
Osteogenesis signal and mineralization of MC3T3-E1 preosteoblasts with various gold plasmonic blackbody (AuPB) contents of AuPB–polycaprolactone (PCL) scaffolds by (a) alkaline phosphate staining and (b) alizarin red S staining (*n* = 5 per group; *p < 0.05; **p < 0.01; and ***p < 0.005; N.S., not significant).

Calcium deposition, a key marker of late-stage osteoblast differentiation and bone matrix mineralization, was analyzed using red staining in Figure 8b. Similar to ALP expression, the ImageJ analysis demonstrated that irradiated groups incorporating AuPBs in PCL scaffolds exhibited significantly greater calcium deposition than the nonirradiated groups. Notably, the 3% AuPB group displayed the highest calcium deposition, with a normalized stained area reaching 180% relative to the nonirradiated group. This result suggests that 3% AuPBs provided an optimal photothermal environment, maintaining temperatures within the subheat shock zone (39–41 °C), which is known to enhance osteoblast function without inducing cellular stress. In contrast, although the 5% AuPB group showed elevated ALP activity, its calcium deposition was slightly reduced. This discrepancy suggests that excessive thermal exposure may have selectively impaired the late-stage mineralization process, highlighting the stage-dependent sensitivity to heat during osteogenesis. This aligns with previous studies indicating that moderate hyperthermia enhances mineralization, whereas excessive heat may disrupt cellular functions.

Collagen-1 is a primary structural component of the bone ECM and plays a crucial role in osteogenesis. Figure 9a presents collagen-1 expression, visualized in green. Quantification using ImageJ software revealed that in the nonirradiated groups (Figure 9b), the 2% AuPB group exhibited the highest collagen-1 expression, with a normalized stained area exceeding 500% compared to the PCL control group. However, under irradiated conditions, the 3% AuPB group demonstrated the highest collagen-1 response, with a normalized stained area exceeding 700% compared to the nonirradiated group. These observations further support the idea that mild photothermal conditions promote ECM synthesis, while excessive heating—such as that potentially induced by 5% AuPBs—may disrupt collagen production due to thermal stress. This further supports the notion that the subheat shock zone (39–41 °C) optimally enhances osteoblastic activity, leading to increased collagen synthesis and improved ECM deposition.

**Figure 9:**
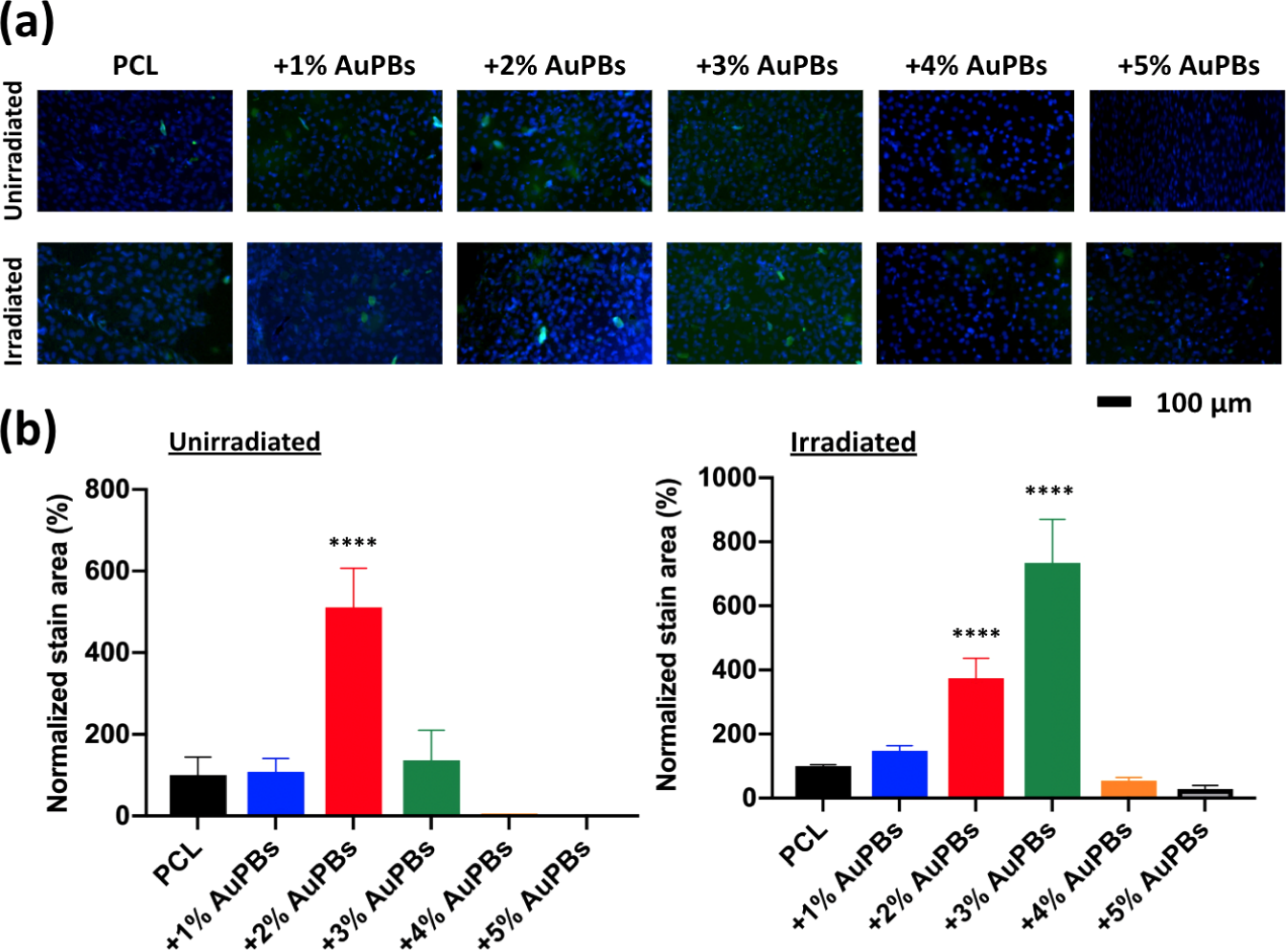
Osteogenic response of MC3T3-E1 preosteoblasts on gold plasmonic blackbody (AuPB)–polycaprolactone (PCL) scaffolds with various AuPB contents. (a) Immunofluorescence staining of collagen-1 expression (green) and cell nuclei (blue, DAPI) after 7 days of culture under unirradiated and irradiated conditions. (b) Quantification of normalized collagen-1-stained area across different groups. The 2% AuPB scaffold showed the highest collagen expression in the unirradiated group, while the 3% AuPB group exhibited the strongest response under irradiation (*n* = 5 per group; ***p < 0.005 and ****p < 0.001; N.S., not significant).

These findings are consistent with previous research demonstrating that moderate hyperthermia promotes mineralization, while excessive heat can impair cellular function. This underscores the importance of precise temperature regulation in PTT to enhance bone formation while preventing cellular stress. However, uncontrolled hyperthermia can have detrimental effects on cellular function, primarily through mitochondrial dysfunction. Elevated temperatures increase mitochondrial membrane permeability, resulting in the loss of membrane potential and impaired oxidative phosphorylation, ultimately leading to reduced ATP production.^65^ Additionally, hyperthermia induces excessive reactive oxygen species (ROS) generation, contributing to oxidative stress and cellular damage.^66^ Mitochondrial respiratory chain complexes, particularly complex I, are highly susceptible to heat-induced dysfunction, further compromising energy metabolism.^67,68^ At extreme temperatures (≥43 °C), mitochondrial stress triggers apoptosis via cytochrome c release, a mechanism commonly exploited in hyperthermia-based cancer therapies.^69^ However, severe ATP depletion shifts cells toward necrotic cell death, leading to inflammation and tissue damage.^70^ These results highlight that while photothermal stimulation can promote osteogenesis, maintaining the temperature within a specific range is essential to avoid impairing different stages of bone formation through mitochondrial damage or oxidative stress. Future studies should focus on long-term cellular responses and *in vivo* bone regeneration to further establish the therapeutic potential of AuPB–PCL scaffolds.

## 4 Conclusions

In this study, we demonstrated that AuPB–PCL scaffolds provide a 3D-printable, mechanically stable, and photothermally responsive platform for bone tissue engineering. The excellent printability of AuPB–PCL composites ensures the fabrication of precisely structured scaffolds with controlled porosity, which is essential for cell infiltration, nutrient diffusion, and tissue integration. Incorporating AuPBs significantly enhanced scaffold mechanical properties while maintaining print fidelity, enabling controlled hyperthermia (39–41 °C) under NIR laser irradiation. Among the tested concentrations, the 3% AuPB group exhibited the most favorable balance across all evaluations, including high cell viability, strong collagen-1 and calcium deposition, and sufficient mechanical performance. This mild thermal stimulation promoted osteoblast viability, cytoskeletal reorganization, ALP activity, and mineralization, optimizing the osteogenic microenvironment. However, excessive heating (>42.5 °C) disrupted mitochondrial function, leading to oxidative stress and impaired cell survival. Therefore, thermal modulation and nanoparticle concentration must be precisely controlled to avoid cytotoxic effects and ensure optimal osteogenic outcomes. These findings underscore the importance of precise temperature modulation in photothermal bone therapies. The combination of high-resolution 3D printing with photothermally active nanomaterials offers a promising strategy for customized, noninvasive bone regeneration solutions. Future studies should explore *in vivo* applications, long-term effects, and potential clinical translation to further validate the therapeutic potential of AuPB–PCL scaffolds for personalized bone repair. In future clinical applications, accurate temperature regulation will be critical to avoid heat-induced cellular damage. This could potentially be achieved using real-time thermal feedback systems, such as infrared thermal cameras or implantable microsensors, to monitor and maintain local temperatures within therapeutic ranges during laser-based photothermal therapy. However, due to the limited penetration depth of NIR laser irradiation, this approach is currently more suitable for treating superficial bone defects, such as those in the tibia, fingers, or joints.
